# Pediatric fever in neutropenia with bacteremia—Pathogen distribution and *in vitro* antibiotic susceptibility patterns over time in a retrospective single-center cohort study

**DOI:** 10.1371/journal.pone.0246654

**Published:** 2021-02-12

**Authors:** Melina Stergiotis, Roland A. Ammann, Sara Droz, Christa Koenig, Philipp Kwame Abayie Agyeman

**Affiliations:** 1 Division of Pediatric Hematology/Oncology, Department of Pediatrics, Inselspital, Bern University Hospital, University of Bern, Bern, Switzerland; 2 Kinderaerzte Kurwerk, Burgdorf, Switzerland; 3 Institute for Infectious Diseases, University of Bern, Bern, Switzerland; 4 Department of Pediatrics, Inselspital, Bern University Hospital, University of Bern, Bern, Switzerland; Universidade de Lisboa Faculdade de Medicina, PORTUGAL

## Abstract

**Background:**

Fever in neutropenia (FN) is a potentially life-threatening complication of chemotherapy in pediatric cancer patients. The current standard of care at most institutions is emergency hospitalization and empirical initiation of broad-spectrum antibiotic therapy.

**Methods:**

We analyzed in retrospect FN episodes with bacteremia in pediatric cancer patients in a single center cohort from 1993 to 2012. We assessed the distribution of pathogens, the *in vitro* antibiotic susceptibility patterns, and their trends over time.

**Results:**

From a total of 703 FN episodes reported, we assessed 134 FN episodes with bacteremia with 195 pathogens isolated in 102 patients. Gram-positive pathogens (124, 64%) were more common than Gram-negative (71, 36%). This proportion did not change over time (p = 0.26). Coagulase-negative staphylococci (64, 32%), viridans group streptococci (42, 22%), *Escherichia coli* (33, 17%), *Klebsiella* spp. (10, 5%) and *Pseudomonas aeruginosa* (nine, 5%) were the most common pathogens. Comparing the *in vitro* antibiotic susceptibility patterns, the antimicrobial activity of ceftriaxone plus amikacin (64%; 95%CI: 56%-72%), cefepime (64%; 95%CI 56%-72%), meropenem (64%; 95%CI 56%-72), and piperacillin/tazobactam (62%; 95%CI 54%-70%), respectively, did not differ significantly. The addition of vancomycin to those regimens would have increased significantly *in vitro* activity to 99% for ceftriaxone plus amikacin, cefepime, meropenem, and 96% for piperacillin/tazobactam (p < 0.001).

**Conclusions:**

Over two decades, we detected a relative stable pathogen distribution and found no relevant trend in the antibiotic susceptibility patterns. Different recommended antibiotic regimens showed comparable *in vitro* antimicrobial activity.

## Introduction

The most frequent potentially lethal complication of chemotherapy in pediatric patients with cancer is fever in neutropenia (FN) [1]. The current management with emergency hospitalization and empirical administration of intravenous broad-spectrum antibiotics has decreased mortality to below 1% in pediatric FN episodes [2]. Bacteremia is detected in approximately every fourth FN episode [1, 3]. Over the last decades, predominantly Gram-positive pathogens have been isolated in FN with bacteremia, likely because of increased use of antibiotic prophylaxis, introduction of long-indwelling intravascular devices, high-dose chemotherapy-induced mucositis, and use of antacids and histamine blockers [4]. Still, because of the potentially devastating course of Gram-negative bacteremia, especially *Pseudomonas aeruginosa*, broad coverage of Gram-positive and Gram-negative pathogens is recommended for initial empirical antibiotic therapy in FN [5]. Because of lower toxicity, and advantages in administration and price, antibiotic monotherapy with an antipseudomonal beta-lactam or a carbapenem is usually recommended by recent European [5–7] and American [8] guidelines, which are based on systematic reviews of observational and randomized trials. A second Gram-negative agent or glycopeptide may be added in patients who are clinically unstable, if an infection with resistant bacteria is suspected, or at centers with a high rate of resistant pathogens [5–8]. Antibiotic resistance has been associated with prolonged bacteremia, increased length of hospitalization and worse outcome in children with cancer and FN [9, 10].

This study aimed to examine the distribution of pathogens isolated during FN with bacteremia, their *in vitro* antibiotic susceptibility patterns against selected antibiotics, and their trends over time in a single center retrospective cohort study spanning two decades in a pediatric oncology center using only oral trimethoprim/sulfamethoxazole or inhaled pentamidine diisethionate antimicrobial prophylaxis.

## Material and methods

### Study design and patients

This retrospective single-center cohort study was conducted at the Division of Pediatric Hematology and Oncology, Department of Pediatrics, Inselspital, Bern University Hospital, University of Bern, Switzerland. All children and adolescents less than 17 years old at diagnosis of malignancy and treated with chemotherapy from 1993 to 2012 were eligible. Details of patient identification and the data acquisition process have been published elsewhere [11]. For this analysis, we only included patients with FN and bacteremia. Information on pathogens isolated in blood culture and *in vitro* antibiotic susceptibility patterns were available from the laboratory database of the local Institute for Infectious Diseases. During the anonymization process the study time period was divided into 4-year intervals (1993 to 1996, 1997 to 2000, 2001 to 2004, 2005 to 2008, 2009 to 2012) [11]. This study was approved by the Institutional Review Board (Direktion Lehre und Forschung, Inselspital Bern; registration number, 13-06-11; last update, April 02, 2014), including waiver of informed consent. On June 30, 2014, data were fully anonymized before analysis, in order to comply with the request of the new Swiss Federal Law on Human Research. Other aspects of subsets of these data have been published before, specifically, data covering the years 2004 to 2007 [1, 12–15], and 2004 to 2011 [2, 16].

### Clinical management

Most patients were treated for their malignancy according to established international phase III protocols or therapy recommendations. Clinical management regarding prophylaxis and treatment of FN remained unchanged during the complete study period. All patients received oral trimethoprim/sulfamethoxazole as primary prophylaxis for *Pneumocystis jiroveci*. Patients who did not tolerate oral trimethoprim/sulfamethoxazole were switched to inhaled pentamidine *diisethionate*. No other antibiotic prophylaxis was used [11]. Routine management of patients with cancer presenting with FN included emergency hospitalization and empiric broad-spectrum intravenous antimicrobial therapy with ceftriaxone plus amikacin as first-line therapy. Hemodynamically unstable patients at presentation of FN were treated with cefepime and amikacin, vancomycin was added if a central venous catheter was present. In patients with FN persisting for more than 48 hours, and in whom no cause of FN had been identified, empirical therapy was usually escalated to meropenem and vancomycin [14]. Aerobic and anaerobic blood cultures were sampled at presentation with FN before starting antimicrobial therapy. Subsequently, one pair of aerobic and anaerobic blood cultures was taken every 24 hours as long as fever continued or in the presence of shaking chills. Blood cultures were collected from each lumen of an existing central venous catheter or peripheral venous line in patients that did not have a central venous catheter [14].

### Definitions

Neutropenia was defined as an absolute neutrophil count ≤ 0.5 G/L [11]. Until July 8, 2007, fever was defined as an axillary temperature ≥ 38.5°C persisting ≥ 2 hours, or a single axillary temperature ≥ 39.0°C, thereafter it was defined as a single tympanic temperature ≥ 39.0°C [2, 11]. In the setting of rising temperatures, the different limits used for the different measurement methods have been shown to be comparable [17]. Intensity of chemotherapy was described using four group classification according to the severity of myelosuppression and the expected duration of severe neutropenia, as described previously [11, 18], which is an extension of an earlier classification with two groups [19]. Several FN episodes per patient were allowed [11]. Bacteremia was defined by the growth of bacteria in blood culture, irrespective of the bacterial species. Blood cultures were analyzed by a qualitative automated culture system (BacT/ALERT^®^, bio-Mérieux, Geneva, Switzerland) [2]. We used the Kirby-Bauer disk diffusion test to determine *in vitro* antibiotic susceptibility. To determine the penicillin and ceftriaxone minimum inhibitory concentrations of viridans group streptococci (VGS) we used the Etest^®^ (bioMérieux, previously AB Biodisk). For *in vitro* susceptibility testing of vancomycin in *Enterococcus* spp. a brain heart infusion agar screen plate containing six micrograms of vancomycin per milliliter was used. During the entire study period, the Clinical and Laboratory Standards Institute standards were used at our Institute for Infectious Diseases [16]. Polymicrobial growth was defined as isolation of different pathogens at the same day of FN episode. If a pathogen was detected in blood cultures taken after the day of FN diagnosis (day 0), this was defined as subsequent bacteremia. Subsequent bacteremia was further subclassified into prolonged bacteremia if the same pathogen was repeatedly detected in blood cultures taken on different days.

#### *In vitro* antibiotic susceptibility patterns

We assessed the *in vitro* antibiotic susceptibility patterns of bacteria isolated in blood culture against antibiotics commonly used for empirical therapy in pediatric FN [5]. We evaluated ceftriaxone plus amikacin, the standard empirical therapy at the study center, cefepime, meropenem, and piperacillin/tazobactam monotherapy, as well as the combinations of above therapy regimens with vancomycin. We considered an antibiotic to be active *in vitro* if the susceptibility pattern showed full susceptibility to the selected antibiotic and the isolated pathogen did not harbor intrinsic resistance to the selected antibiotic. We considered combination therapy (e.g. ceftriaxone plus amikacin) to be active *in vitro* if the susceptibility pattern showed full susceptibility to at least one of the antibiotics and did not harbor intrinsic resistance against all antibiotics of combination therapy. *Enterococci* spp. were considered intrinsic resistant to cephalosporins and carbapenems.

Due to technical difficulties in retrieving data from the microbiology database, results of susceptibility testing were not available from 1993 to mid 1998. Therefore, we excluded the time period from 1993 to 1996 and all other isolates without available susceptibility testing from analysis of *in vitro* antibiotic susceptibility patterns.

### Statistical analysis

We presented continuous variables as median and interquartile range (IQR) and nominal data as frequencies with 95% confidence intervals (95% CI). We used the exact Kruskal-Wallis Test to analyze trends over time for nominal data and exact Jonckheere-Terpstra-Test for continuous data with Monte Carlo approximation. We compared the *in vitro* antibiotic susceptibility patterns with the Fisher’s Exact Test.

For all statistical analyses, 2-sided P-values below 0.05 were considered significant. The exact Kruskal-Wallis test and exact Jonckheere-Terpstra test were calculated with StatXact 10 (Cytel Inc., Cambridge, MA USA). All other analyses and plots were done using R 3.3.3 (R Foundation for Statistical Computing, Vienna Austria).

## Results

### Patients and FN episodes

**Fig 1:**
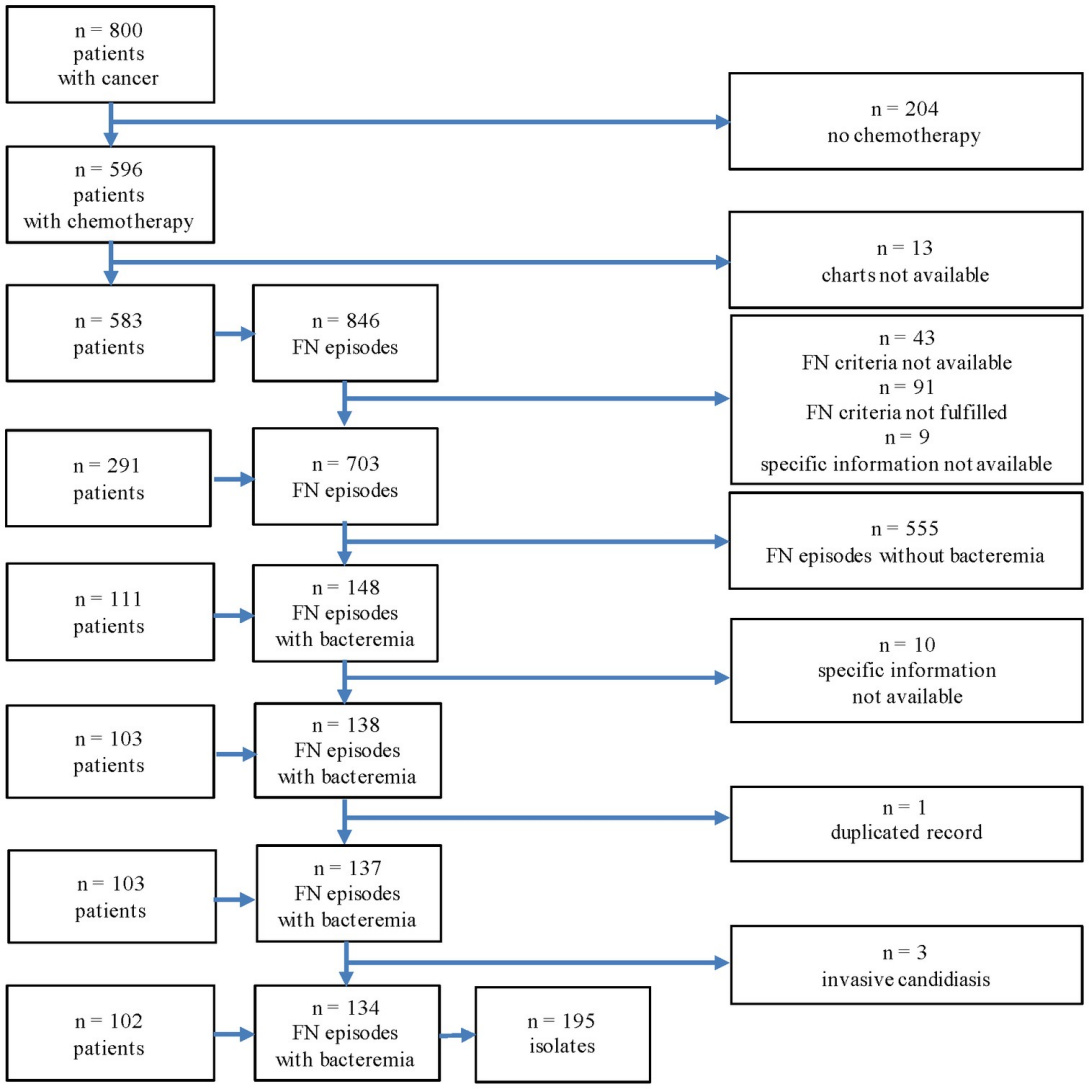
Flowchart of FN episodes in pediatric patients with cancer and fever in neutropenia, from 1993 to 2012. Multiple FN episodes per patient allowed. FN = fever and neutropenia.

In the 20 study years, 800 pediatric patients less than 17 years old with cancer were treated at the University Children‘s Hospital Bern. 596 (75%) received chemotherapy to treat their malignancy. Of 703 eligible FN episodes, bacteremia was detected in 148 (21%). Specific information on the clinical course was available in 138 (90%) of these episodes [11]. Four (3%) episodes were excluded from analysis because of duplicate information in one and blood-culture proven invasive fungal infection due to *Candida* spp. in three. We thus analyzed 134 FN episodes with bacteremia caused by 195 bacteria isolated in blood culture in 102 patients (Fig 1). In total 18 of 102 (18%) patients were registered with two and seven (7%) patients with three bacteremia episodes.

### Clinical characteristics of FN episodes with bacteremia

The proportion of FN episodes with bacteremia did not change over time (p = 0.91). Clinical characteristics and evolution over time of the 134 FN episodes with bacteremia are presented in Table 1. Over time, we observed less FN episodes in patients with relapse of malignancy. In 107 of 133 (80%) episodes the combination of ceftriaxone plus amikacin was administered as initial antibiotic treatment. Switch of antibiotic treatment could include a de-escalation to a specific treatment based on the susceptibility testing or escalation to broaden the antibiotic spectrum like meropenem plus vancomycin. A potentially life-threatening complication (not otherwise specified) occurred in eight (6%) episodes, in seven (5%) patients were admitted to the intensive care unit, and in two (1%) children died. Among patients treated on intensive care unit Gram-negative sepsis with E. coli was found in three (3 of 7, 43%) patients (S1 Table). CoNS bacteremia was detected in one child that died, however, the isolate was sensitive to ceftriaxone and was only isolated in one blood culture. He finally succumbed to invasive fungal infection due to *Aspergillus fumigatus*. The other child died on day 3 with fulminant VGS sepsis detected on day 0. He received antibiotic treatment with ceftriaxone and amikacin without treatment escalation, susceptibility testing of the isolate was not performed.

**Table 1:** Characteristics of FN episodes with bacteremia in pediatric patients with cancer, from 1993 to 2012.

		Time period					P value^h^
FN episodes with bacteremia of all FN episodes ^a^	All	1993–1996	1997–2000	2001–2004	2005–2008	2009–2012	
	134 of 703 (19%)	19 of 138 (14%)	29 of 137 (21%)	40 of 167 (24%)	32 of 153 (21%)	14 of 108 (13%)	0.91
Patient related characteristics							
Female	67 (50%)	6 (32%)	15 (52%)	19 (48%)	20 (62%)	7 (50%)	0.13
Age at FN diagnosis^b^							0.054
0–3 years	42 (31%)	6 (32%)	5 (17%)	12 (30%)	12 (38%)	7 (50%)	
4–7 years	22 (16%)	2 (11%)	5 (17%)	3 (8%)	9 (28%)	3 (21%)	
8–11 years	30 (22%)	3 (16%)	12 (41%)	9 (22%)	4 (12%)	2 (14%)	
12–17 years	40 (30%)	8 (42%)	7 (24%)	16 (40%)	7 (22%)	2 (14%)	
Disease related characteristics							
Diagnostic group^b^^,^^c^							0.12
Acute lymphoblastic leukemia	67 (50%)	11 (58%)	17 (59%)	15 (38%)	18 (56%)	6 (43%)	
Acute myeloid leukemia	26 (19%)	2 (11%)	2 (7%)	9 (22%)	9 (28%)	4 (29%)	
Non-Hodgkin lymphoma	14 (10%)	1 (5%)	5 (17%)	6 (15%)	1 (3%)	1 (7%)	
Solid tumors outside of CNS	20 (15%)	4 (21%)	5 (17%)	7 (18%)	2 (6%)	2 (14%)	
Tumors of CNS	7 (5%)	1 (5%)	0	3 (8%)	2 (6%)	1 (7%)	
Relapse	29 (22%)	8 (42%)	9 (31%)	4 (10%)	5 (16%)	3 (21%)	0.030
Bone marrow involvement	39 (29%)	7 (37%)	5 (17%)	14 (35%)	10 (31%)	3 (21%)	0.98
Therapy related characteristics							
Chemotherapy^b^^,^^d^							0.49
Intensity 1	7 (5%)	0	4 (14%)	2 (5%)	1 (3%)	0	
Intensity 2	91 (68%)	16 (84%)	17 (59%)	28 (70%)	22 (69%)	8 (57%)	
Intensity 3	34 (25%)	3 (16%)	7 (24%)	10 (25%)	9 (28%)	5 (36%)	
Intensity 4	2 (1%)	0	1 (3%)	0	0	1 (7%)	
Central venous access device	89 (66%)	11 (58%)	18 (62%)	26 (65%)	21 (66%)	13 (93%)	0.09
Initial intravenous antibiotic treatment with ceftriaxone + amikacin^e^	107 (80%)	17 (89%)	25 (86%)	32 (80%)	22 (71%)	11 (79%)	0.08
Switched intravenous antibiotic treatment	60 (45%)	1 (5%)	3 (10%)	24 (60%)	23 (72%)	9 (64%)	ND^i^
Antibiotic treatment at discharge^d^	37 (28%)	9 (47%)	2 (7%)	10 (26%)	11 (34%)	5 (36%)	0.51
Duration of intravenous antibiotic treatment (days)^d^	10 (9–13)	11 (9–14)	10 (10–11)	11 (10–15)	11 (10–14)	9 (8–11)	0.97^i^
Duration of total (intravenous + oral) antibiotic treatment (days)^d^	10 (9–14)	11 (9–14)	10 (10–11)	12 (10–15)	11 (10–14)	9 (9–11)	0.92^i^
Duration of hospitalisation (days)	10 (5–13)	10 (8–14)	7 (3–10)	12 (5–14)	10 (6–14)	10 (6–14)	0.042^i^
Bacteremia							
Polybacterial growth^f^	15 (11%)	0	8 (28%)	3 (8%)	4 (12%)	0	0.40
Subsequent bacteremia, any time^g^	46 (34%)	11 (58%)	8 (28%)	17 (42%)	7 (22%)	3 (21%)	0.031
Subsequent bacteremia, day 1 or 2^g^	35 (26%)	9 (47%)	8 (28%)	13 (32%)	5 (16%)	0	0.002
Subsequent bacteremia, beyond day 2^g^	16 (12%)	3 (16%)	0	7 (18%)	3 (9%)	3 (21%)	0.39

Categorical variables are presented as frequency (%) and continuous variables as median (IQR = interquartile range). Column percentages are presented; percentages are based on available data for each variable. FN = fever and neutropenia. CNS = central nervous system. ND = not done.

^a^Data of all FN episodes [11].

^b^Percentages do not sum up to 100% because of rounding differences.

^c^No FN with bacteremia in patients with Hodgkin lymphoma.

^d^Chemotherapy intensity was classified in four groups according the severity of myelosuppression to the expected duration of severe neutropenia, the higher the number the longer.

^e^Data not available for 1 episode.

^f^Polybacterial growth was defined as isolation of different pathogens at the same day of FN episode.

^g^Subsequent bacteremia was defined, if pathogens were detected in an episode after day 0. Subsequent bacteremia at different time points per episodes possible, numbers do not add up.

^h^Exact Kruskal-Wallis-Test (Monte-Carlo approximation) for analyses of trend over time.

^i^The coding of switch of antibiotics changed between the period before 2000 and after. Therefore we have not performed statistical analysis.

### Pathogens in FN episodes

124 (64%, 95% CI 56%-70%) Gram-positive and 71 (36%; 95% CI 30%-44%) Gram-negative bacteria were isolated in blood culture (Table 2). This proportion did not change over time (p = 0.26), but we observed a significant change over time in Gram-negative pathogen distribution (p = 0.021), mainly due to an increased detection of *Klebsiella* spp. during the last time period. (Table 2). If we excluded CoNS, pathogen distribution changed to more Gram-negative 71 (54%; 95% CI 45%-62%) than Gram-positive 61 (46%; 95% CI 38%-55%) bacteria (S2 Table).

**Table 2:** Bacteria detected in blood culture, from 1993 to 2012.

	Time period						P value^c^
	All isolates	1993–1996	1997–2000	2001–2004	2005–2008	2009–2012	
	n = 195	n = 26	n = 46	n = 61	n = 43	n = 19	
Gram-positive bacteria	124 (64%)	21 (81%)	25 (54%)	42 (69%)	26 (60%)	10 (53%)	0.26
CoNS	63 (32%)	10 (38%)	10 (22%)	28 (46%)	9 (21%)	6 (32%)	0.77
*S*. *aureus*	5 (3%)	3 (12%)	0	0	2 (5%)	0	
VGS	42 (22%)	5 (19%)	11 (24%)	11 (18%)	13 (30%)	2 (11%)	
*Enterococcus* spp.	5 (3%)	1 (4%)	1 (2%)	3 (5%)	0	0	
Other Gram-positive^a^	9 (5%)	2 (8%)	3 (7%)	0	2 (5%)	2 (11%)	
Gram-negative bacteria	71 (36%)	5 (19%)	21 (46%)	19 (31%)	17 (40%)	9 (47%)	
*E*. *coli*	33 (17%)	4 (15%)	12 (26%)	7 (11%)	7 (16%)	3 (16%)	0.021
*Klebsiella* spp.	10 (5%)	0	1 (2%)	4 (7%)	1 (2%)	4 (21%)	
*P*. *aeruginosa*	9 (5%)	0	0	4 (7%)	5 (12%)	0	
*Enterobacter* spp.	6 (3%)	0	4 (9%)	2 (3%)	0	0	
Other Gram-negative^b^	19 (10%)	1 (4%)	8 (17%)	4 (7%)	4 (9%)	2 (11%)	

Data are frequency (%). Column percentages are presented; percentages are based on available data for each variable. CoNS = coagulase-negative staphylococci. VGS = Viridans group streptococci.

^a^Streptococcus spp. (3), *Bacillus* spp. (2), *Corynebacterium* sp. (1), *Granulicatella adjacens* (1), gram-positive cocci in chains, not otherwise specified (1), *Micrococcus kristinae* (1).

^b^*Capnocytophaga* spp. (6), *Haemophilus influenzae* (2), *Neisseria* spp. (2), *Acinetobacter lwoffii* (1), *Fusobacterium nucleatum* (1), *Moraxella osloensis* (1).

^c^Exact Kruskal-Wallis (Monte-Carlo approximation) test for analyses of trend over time.

The majority of pathogens, 134 of 195 (69%) were detected on day 0 only. All 71 Gram-negative bacteria were isolated in blood cultures taken before day 3, 61 (86%) in the first blood culture taken on day 0, nine (13%) on day 1, and one (1%) on day 2.

**Fig 2:**
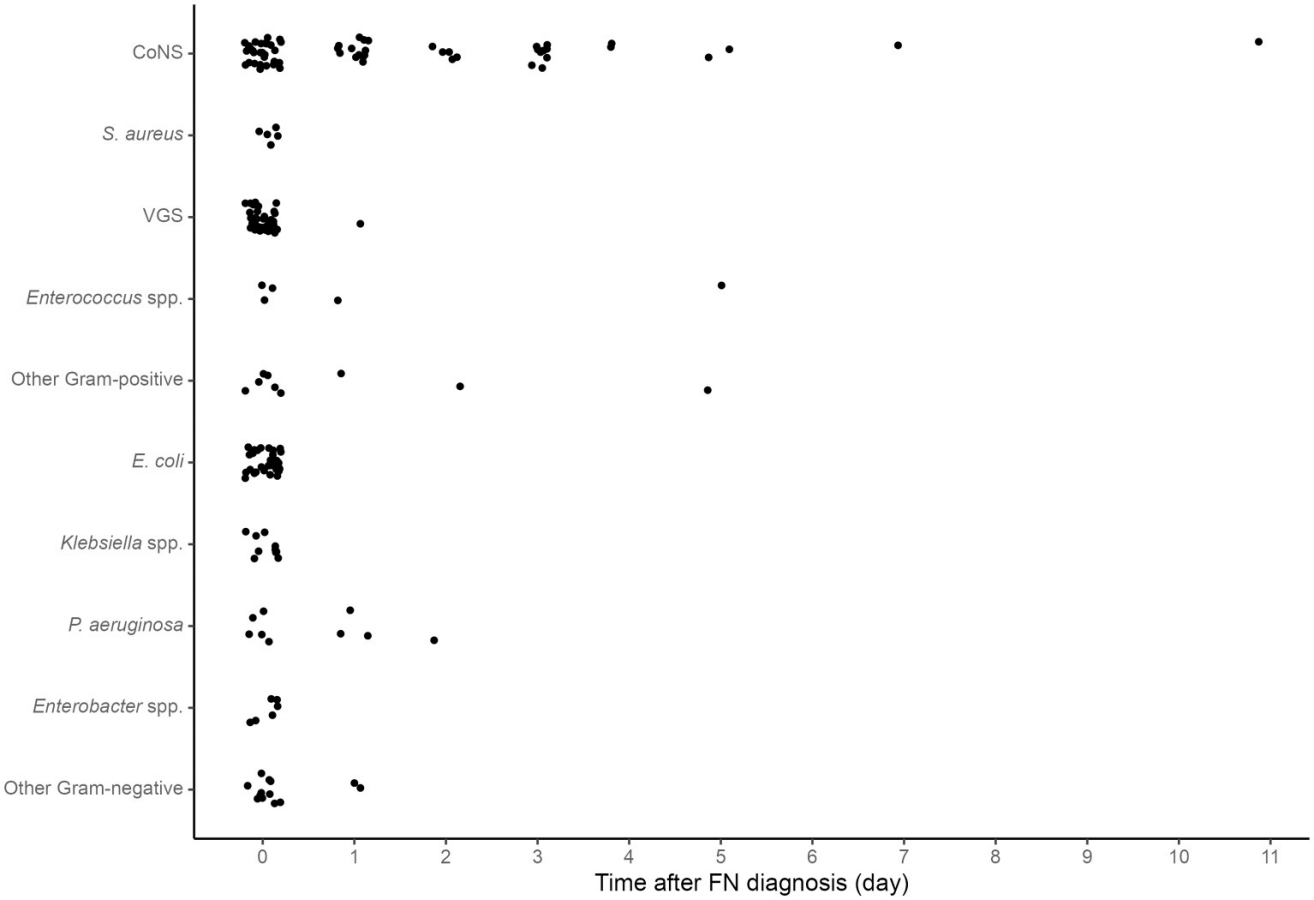
Temporal distribution of bacteria detection in pediatric patients with cancer and fever in neutropenia. Each point represents one isolated pathogen (n = 195) on the day when the blood culture was taken after FN diagnosis. All positive blood cultures taken during FN episode are shown. FN = fever in neutropenia. CoNS = coagulase-negative staphylococci. VGS = Viridans group streptococci. Day 0 = day of FN diagnosis. Day 1 = the following calendar day after FN diagnosis and so forth.

Subsequent bacteremia was detected in 46 of 134 (34%) episodes (Table 1). All 20 bacteria detected in blood cultures sampled after day 2 were Gram-positive, 17 CoNS, one *Enterococcus faecalis* and two other Gram-positive pathogens (Fig 2). Over time, fewer episodes with subsequent bacteremia at any time and day 1 or 2 were detected (p = 0.031 and p = 0.002, respectively, Table 1). This time trend remained significant when only considering CoNS if there was prolonged CoNS bacteremia (28 of 111 (25%), p < 0.001) or when only considering bacteria isolated before day 3 and excluding all CoNS (18 of 101 (18%), p = 0.002, details not shown).

Prolonged bacteremia was detected in 12 of 134 episodes (9%), caused by CoNS in 10 and *P*. *aeruginosa* in two episodes, none had a fatal outcome. In 15 (11%) episodes polymicrobial growth was noted in blood culture.

### *In vitro* antibiotic susceptibility patterns against selected antibiotic therapy regimens

**Fig 3:**
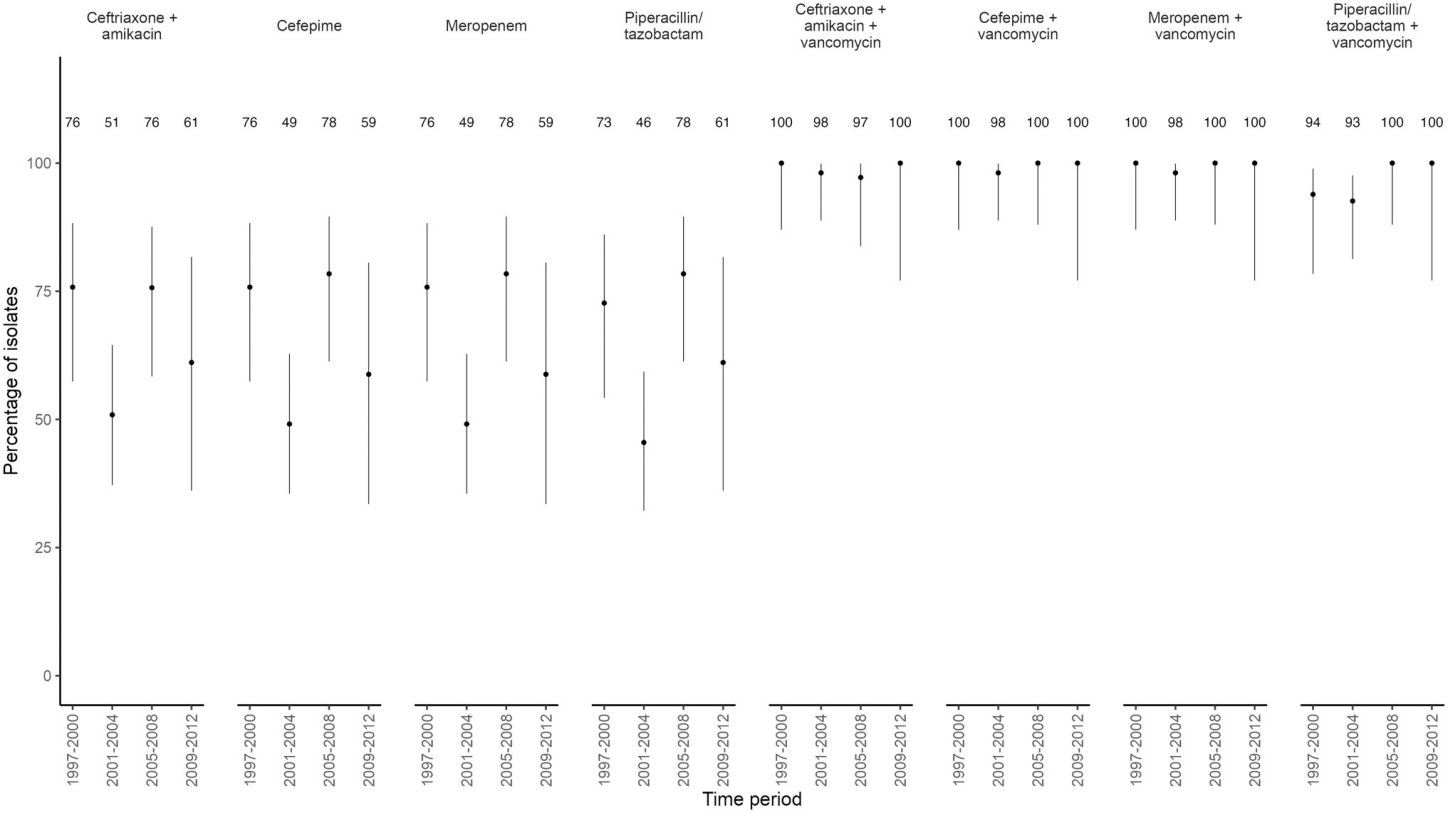
*In vitro* antibiotic susceptibility patterns of empirical antibiotic therapy in bacteria detected in blood culture, from 1997 to 2012. Points indicate the point estimate for the percentage of *in vitro* antibiotic susceptibility for each time period in 143 of 169 isolates with full antimicrobial susceptibility information. Vertical lines present the 95% CI`s around the point estimates.

We analyzed *in vitro* antibiotic susceptibility patterns of 143 of 169 (85%) bacteria isolated in blood culture between 1997 and 2012. The combination of ceftriaxone plus amikacin was active *in vitro* in 64% (92 of 143; 95% CI 56%-72%), cefepime 64% (91 of 142; 95% CI 56%-72%), meropenem 64% (91 of 142; 95% CI 56%-72%), and piperacillin/tazobactam 62% (89 of 143; 95% CI 54%-70%). Adding vancomycin to any empirical antibiotic therapy would have increased activity to 99% for ceftriaxone plus amikacin (138 of 140; 95% CI: 94%-100%), cefepime (139 of 140; 95% CI: 96%-100%), and meropenem (139 of 140; 95% CI: 96%-100%), while it would have increased activity to 96% for piperacillin/tazobactam (134 of 140; 95% CI: 91%-98%) (p < 0.001 for all comparisons). No significant trend over time was noted (Fig 3).

The isolates not covered *in vitro* by ceftriaxone plus amikacin were mostly Gram-positive bacteria (51 of 52, 98%; 44 CoNS, 4 *Enterococcus* spp., 3 VGS) and one Gram-negative pathogen (*P*. *aeruginosa* isolate resistant to Amikacin). None of the respective patients died.

If we excluded CoNS fully sensitive to vancomycin, the antibiotic *in vitro* susceptibility rates for the examined antibiotic regimens were increased to 89% for piperacillin/tazobactam, 92% for ceftriaxone plus amikacin, cefepime, meropenem. In combination with vancomycin in vitro susceptibility rates reached 93% for piperacillin/tazobactam, 98% for ceftriaxone plus amikacin and 99% for cefepime and meropenem (S1 Fig). No significant trend over time was noted. Piperacillin/tazobactam was tested *in vitro* resistant to five *E*. *coli* and one *Klebsiella pneumoniae* isolates.

In the study period, we did not detect methicillin-resistant *Staphylococcus aureus* or extended-spectrum beta-lactamase (ESBL)-producing *Enterobacteriaceae*. Of note, one vancomycin-resistant *E*. *faecalis* (but sensitive to amoxicillin, teicoplanin, and norfloxacin) and two VGS isolates resistant to ceftriaxone (ceftriaxone E Test ≥ 4 μg/ml) were detected. Among *E*. *coli* and *Klebsiella* spp. 18% and 10%, respectively, were resistant to piperacillin/tazobactam (Table 3).

**Table 3:** Results of *in vitro* antibiotic susceptibility testing in the three most common Gram-positive and Gram-negative bacteria detected in blood culture, from 1997 to 2012.

	Bacteria	Antibiotic	Number of resistant isolates
Gram-positive bacteria	Coagulase-negative staphylococci (n = 53)^a^	Penicillin	49 (96%)
		Oxacillin	44 (86%)
		Gentamycin	22 (43%)
		Tetracycline	11 (22%)
		Trimethoprim/sulfamethoxazole	44 (86%)
		Vancomycin	0
		Clindamycin	32 (63%)
	Viridans group streptococci (n = 37)	Penicillin (E Test ≤ 0.12 μg/ml)^b^	16 (67%)
		Penicillin (E Test ≥ 4.0 μg/ml) ^b^	4 (17%)
		Ceftriaxone (E Test ≤ 1.0 μg/ml)^c^	15 (88%)
		Ceftriaxone (E Test ≥ 4.0 μg/ml)^c^	2 (12%)
	*Enterococcus* spp. (n = 4)	Ampicillin	1 (25%)
		Vancomycin	1 (25%)
		Gentamycin high-level resistance	3 (75%)
		Streptomycin high-level resistance	3 (75%)
Gram-negative bacteria	*E*. *coli* (n = 29)	Ampicillin^d^	24 (86%)
		Amoxicillin/clavulanic acid^d^	19 (68%)
		Cefuroxime^d^	23 (82%)
		Piperacillin/tazobactam^d^	5 (18%)
		Ceftriaxone^d^	0
		Cefepime^e^	0
		Meropenem^f^	0
		Amikacin^d^	0
		Trimethoprim/sulfamethoxazole^d^	24 (86%)
		Ciprofloxacin^d^	1 (4%)
	*Klebsiella* spp. (n = 10)	Amoxicillin/clavulanic acid	3 (30%)
		Cefuroxime	10 (100%)
		Piperacillin/tazobactam	1 (10%)
		Ceftriaxone	0
		Cefepime^d^	0
		Meropenem^d^	0
		Amikacin	1 (10%)
		Trimethoprim/sulfamethoxazole	5 (50%)
		Ciprofloxacin	1 (10%)
	*P*. *aeruginosa* (n = 9)	Piperacillin/tazobactam	0
		Ceftazidime	1 (11%)
		Cefepime	0
		Meropenem	0
		Amikacin	1 (11%)
		Tobramycin	1 (11%)
		Ciprofloxacin^d^	0
	*Enterobacter* spp. (n = 6)	Amoxicillin/clavulanic acid	6 (100%)
		Cefuroxime	6 (100%)
		Piperacillin/tazobactam	0
		Ceftriaxone	0
		Cefepime	0
		Meropenem	0
		Amikacin	0
		Trimethoprim/sulfamethoxazole	4 (67%)
		Ciprofloxacin	0

Data are frequency (%).

^a^Data not available for two isolates.

^b^Data not available for 13 isolates.

^c^Data not available for 20 isolates.

^d^Data not available for one isolate.

^e^Data not available for 6 isolates.

^f^Data not available for 5 isolates.

## Discussion

Over a 20-years observation period in a single tertiary pediatric oncology center, we report a stable distribution of pathogens and antibiotic susceptibility patterns without emergence of multi-resistant pathogens in pediatric patients with cancer diagnosed with FN with bacteremia. Our results can serve as a proof that in a setting with low antimicrobial resistance rates in the general population [20], infections with multi-resistant pathogens remain uncommon in children with cancer.

Comparable to other studies in pediatric patients with cancer and FN [4, 8, 9, 21, 22], we detected more Gram-positive than Gram-negative pathogens in blood culture, and CoNS was the most frequent pathogen detected. CoNS also constituted the main pathogen detected in episodes with subsequent bacteremia. An American study found subsequent bacteremia (day 1–13) only in 4% (four of 95) episodes with persistent fever [23]. Our data support that relevant bacteremia is rarely detected in pediatric patients with cancer and FN after day 3 unless the patient becomes unstable [23]. While the addition of a glycopeptide to empirical antibiotic therapy would have led to a better *in vitro* coverage of isolated bacteria, current guidelines reserve this for selected patients [5, 6, 8]. The addition of a glycopeptide to initial empirical antibiotic therapy regimens in FN in patients with cancer is not associated with reduced duration of fever or a decrease in overall mortality [8], CoNS infections are rarely associated with a severe disease course [24], and the addition of a glycopeptide to initial therapy in FN has been associated with more frequent adverse effects [5].

Current guidelines—which were not available at the time of this study—do favor monotherapy with an antipseudomonal beta-lactam or a carbapenem as first-line treatment [5–8]. In our setting, the *in vitro* antibiotic susceptibility patterns of ceftriaxone plus amikacin was comparable to cefepime, meropenem, and piperacillin/tazobactam (with or without CoNS). However, in one of five episodes with *P*. *aeruginosa* bacteremia susceptibility testing revealed resistance to ceftriaxone and amikacin, whilst the isolate would have been susceptible in vitro to cefepime, meropenem, and piperacillin/tazobactam.

All Gram-negative pathogens detected in our study were isolated in blood cultures taken before day 3, underscoring the need for a broad coverage of Gram-positive and Gram-negative pathogens by empirical therapy regimens for FN in pediatric cancer patients. Similar to other studies, *E*. *coli* was the most prevalent Gram-negative pathogen in our study [22, 25]. While resistance to piperacillin-tazobactam was more prevalent in our cohort (18%, 5 of 28) than in the general pediatric population in a comparable setting (7%, 18 of 275) [20], we did not detect ESBL-producing *Enterobacteriaceae* infections. This is in contrast to other studies in pediatric patients with cancer and FN that have reported increased rates of multi-resistant bacterial infections [9, 10, 22, 25–27].

The strengths of the study are the long observation period and a large number of FN episodes with bacteremia in a setting without relevant changes in clinical management during the study period. To the best of our knowledge, our study is the longest observational study examining the pathogen distribution, the *in vitro* antibiotic susceptibility patterns and their trends over two decades in pediatric cancer patients.

Given the retrospective nature of the study, several limitations need to be mentioned. First, data on *in vitro* antibiotic susceptibility patterns were not available for some pathogens due to a change in the data storage system during the study period. Second, clinical data was incomplete which prevented us from drawing firm conclusions on reasons and nature of adaption of antibiotic therapies and classification of pathogens as contaminants. Third, the use of Kirby-Bauer disk diffusion test might be less sensitive to the detection of changes in susceptibility rates than determining minimum inhibitory concentrations. However, given the long observation period of the study, we do not think this affects the interpretation of the results. Fourth, several FN episodes per patients were allowed and analyses were not formally corrected for multiple episodes per patient.

In conclusion, this study showed a stable local distribution of pathogens causing bacteremia during FN episodes in pediatric cancer patients with FN over a 20-years period. The currently used empirical treatment, with its advantage of once daily application, had an *in vitro* activity that was comparable to other recommended treatment regimens and was stable over time. In our setting, a fourth-generation cephalosporin would be preferred as antipseudomonal monotherapy, given the lack of ESBL-resistance and the relatively high rate of resistance against piperacillin/tazobactam observed in *Enterobacteriaceae*.

## Supporting information

S1 Fig (TIF)

S1 Table Episodes with fatal outcome or need of intensive care unit. (DOCX)

S2 Table Bacteria detected in blood culture, from 1993 to 2012, without coagulase-negative staphylococci. (DOCX)
