# Supplementary figures and images for: Pediatric fever in neutropenia with bacteremia—Pathogen distribution and in vitro antibiotic susceptibility patterns over time in a retrospective single-center cohort study
Source: PLoS One. 2021 Feb 12;16(2):e0246654. doi: 10.1371/journal.pone.0246654 (PMC7880464; doi:10.1371/journal.pone.0246654)

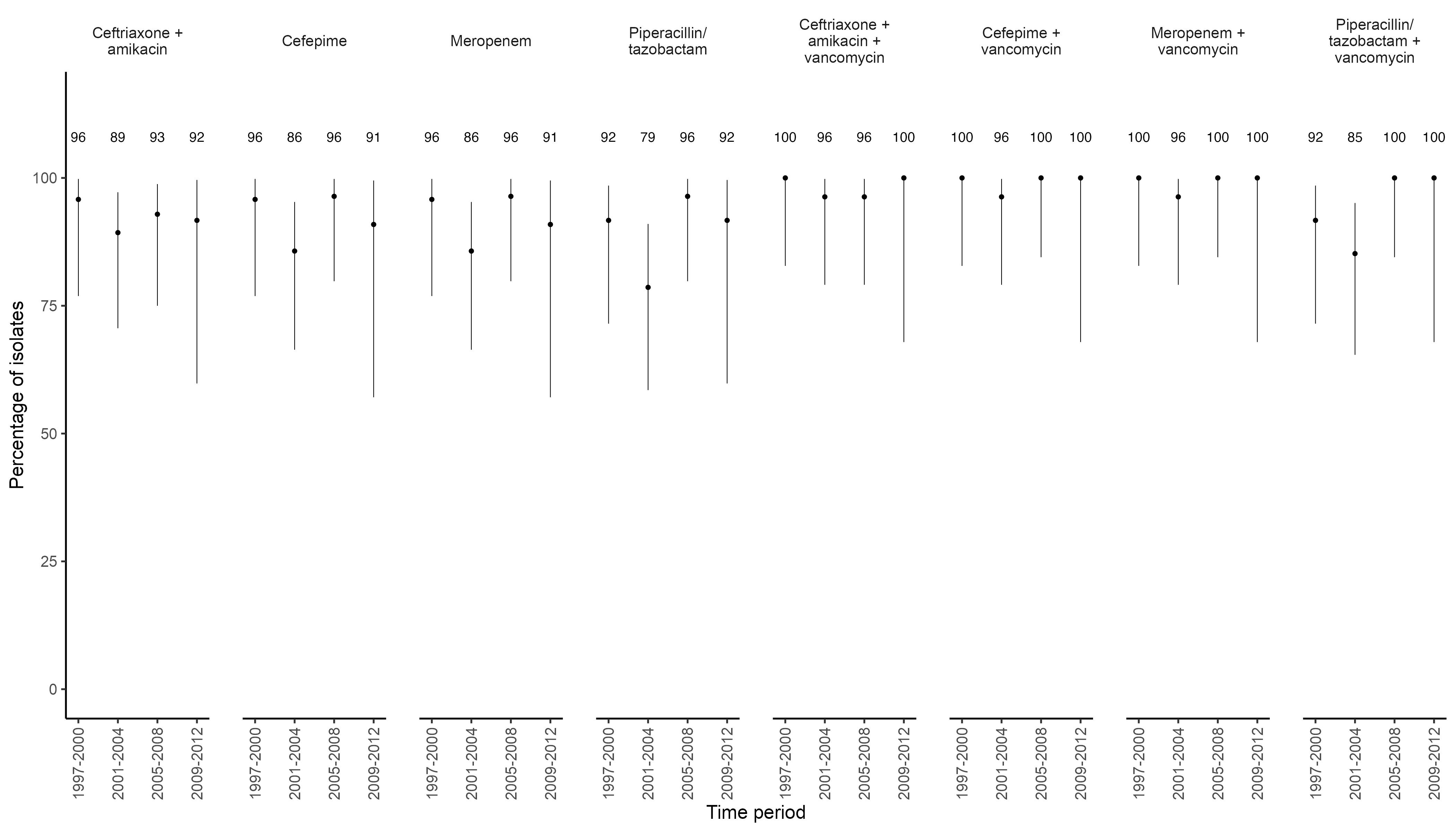

Supplement: S1 Fig — (TIF) [file pone.0246654.s001.tif]
