# Supplementary material for: Pediatric fever in neutropenia with bacteremia—Pathogen distribution and in vitro antibiotic susceptibility patterns over time in a retrospective single-center cohort study
Source: PLoS One. 2021 Feb 12;16(2):e0246654. doi: 10.1371/journal.pone.0246654 (PMC7880464; doi:10.1371/journal.pone.0246654)
Supplement: S1 Table — (DOCX) [file pone.0246654.s002.docx]

**S1 Table Episodes with fatal outcome or need of intensive care unit**

| Time period | Age (years) | Diagnostic Group | Blood culture results | | | Intravenous antibiotic therapy | | | Length of ICU stay | Length of hospital stay | Survival |
| --- | --- | --- | --- | --- | --- | --- | --- | --- | --- | --- | --- |
|  |  |  | Day of detection | Isolated bacteria | Susceptibility testing for CRO + AMK | Initial therapy | Therapy switched | Length of therapy |  |  |  |
| 2001-2004 | 8-11 | AML | 0 | VGS | NA | CRO + AMK | No | 3 | 0 | 2 | Died |
| 2001-2004 | 12-17 | AML | 0 | VGS | S | CRO + AMK | Yes | 16 | 5 | 30 | Survived |
| 2001-2004 | 0-3 | Solid | 0 | CoNS | S | CRO + AMK | Yes | 12 | 26 | 2 | Died |
| 2005-2008 | 12-17 | ALL | 0 | *S. mucilaginosus*, CoNS, *S. aureus* | S | CRO + AMK | Yes | 39 | 9 | 60 | Survived |
| 2005-2008 | 4-7 | ALL | 0 | CoNS | R | Monotherapy | Yes | 23 | 10 | 2 | Survived |
| 2005-2008 | 12-17 | ALL | 0 | *E. coli* | S | Monotherapy | No | NA | 1 | 9 | Survived |
| 2005-2008 | 8-11 | ALL | 0 | *E. coli* | S | CRO + AMK | No | 14 | 4 | 25 | Survived |
| 2005-2008 | 8-11 | NHL | 0 | *E. coli* | S | Combination therapy | Yes | 26 | 26 | 26 | Survived |

AML = acute myeloid leukemia, Solid = solid tumor outside of central nervous system, ALL = acute lymphatic leukemia, NHL = non-Hodgkin lymphoma,
CoNS = coagulase-negative Staphylococci, VGS = Viridans group streptococci, CRO + AMK = ceftriaxone plus amikacin, S = *in vitro* sensitive,
R = *in vitro* resistant, NA = data not available, ICU = intensive care unit
