# Supplementary material for: Pediatric fever in neutropenia with bacteremia—Pathogen distribution and in vitro antibiotic susceptibility patterns over time in a retrospective single-center cohort study
Source: PLoS One. 2021 Feb 12;16(2):e0246654. doi: 10.1371/journal.pone.0246654 (PMC7880464; doi:10.1371/journal.pone.0246654)
Supplement: S2 Table — (DOCX) [file pone.0246654.s003.docx]

**S2 Table: Bacteria detected in blood culture, from 1993 to 2012, without coagulase-negative staphylococci**

|  | Time period | | | | | | P value^c^ |
| --- | --- | --- | --- | --- | --- | --- | --- |
|  | All isolates | 1993-1996 | 1997-2000 | 2001-2004 | 2005-2008 | 2009-2012 |  |
|  | n = 132 | n = 16 | n = 36 | n = 35 | n = 34 | n = 13 |  |
| Gram-positive bacteria | 61 (46%) | 11 (69%) | 15 (42%) | 14 (42%) | 17 (50%) | 4 (31%) | 0.28 |
| *S. aureus* | 5 (4%) | 3 (19%) | 0 | 0 | 2 (6%) | 0 | 0.60 |
| VGS | 42 (32%) | 5 (31%) | 11 (31%) | 11 (33%) | 13 (38%) | 2 (15%) |  |
| *Enterococcus* spp. | 5 (4%) | 1 (6%) | 1 (3%) | 3 (9%) | 0 | 0 |  |
| Other Gram-positive^a^ | 9 (7%) | 2 (12%) | 3 (8%) | 0 | 2 (6%) | 2 (15%) |  |
| Gram-negative bacteria | 71 (54%) | 5 (31%) | 21 (58%) | 19 (58%) | 17 (50%) | 9 (69%) |  |
| *E. coli* | 33 (25%) | 4 (25%) | 12 (33%) | 7 (21%) | 7 (21%) | 3 (23%) | 0.028 |
| *Klebsiella* spp. | 10 (8%) | 0 | 1 (3%) | 4 (12%) | 1 (3%) | 4 (31%) |  |
| *P. aeruginosa* | 9 (7%) | 0 | 0 | 4 (12%) | 5 (15%) | 0 |  |
| *Enterobacter* spp. | 6 (5%) | 0 | 4 (11%) | 2 (6%) | 0 | 0 |  |
| Other Gram-negative^b^ | 13 (10%) | 1 (6%) | 4 (11%) | 2 (6%) | 4 (12%) | 2 (15%) |  |

Data are frequency (%). Column percentages are presented; percentages are based on available data for each variable. VGS = Viridans group streptococci.
^a^Streptococcus spp. (3), *Bacillus* spp. (2), *Corynebacterium* sp. (1), *Granulicatella adjacens* (1), gram-positive cocci in chains, not otherwise specified (1), *Micrococcus kristinae* (1). ^b^*Capnocytophaga* spp. (6)*, Haemophilus influenzae* (2), *Neisseria* spp. (2), *Acinetobacter lwoffii* (1), *Fusobacterium nucleatum* (1), *Moraxella osloensis* (1).
^c^Exact Kruskal-Wallis (Monte-Carlo approximation) test for analyses of trend over time.
